# Long-Term Safety of Level II Oncoplastic Surgery after Neoadjuvant Treatment for Locally Advanced Breast Cancer: A 20-Year Experience

**DOI:** 10.3390/jcm13133665

**Published:** 2024-06-23

**Authors:** Alejandro M. Sanchez, Flavia De Lauretis, Angela Bucaro, Niccolo Borghesan, Chiara V. Pirrottina, Antonio Franco, Lorenzo Scardina, Diana Giannarelli, Jenny C. Millochau, Marina L. Parapini, Alba Di Leone, Fabio Marazzi, Armando Orlandi, Antonella Palazzo, Alessandra Fabi, Riccardo Masetti, Gianluca Franceschini

**Affiliations:** 1 Multidisciplinary Breast Center—Dipartimento Scienze della Salute della Donna e del Bambino e di Sanità Pubblica, Fondazione Policlinico Universitario A. Gemelli IRCCS, 00168 Rome, Italy; martin.sanchez@hotmail.it (A.M.S.); flavia.delauretis@gmail.com (F.D.L.); angela.bucaro@hotmail.com (A.B.); niccolo.borghesan@gmail.com (N.B.); chiaravaleriapirrottina@gmail.com (C.V.P.); antonio.franco89@icloud.com (A.F.); alba.dileone@policlinicogemelli.it (A.D.L.); riccardo.masetti@policlinicogemelli.it (R.M.); gianluca.franceschini@policlinicogemelli.it (G.F.); 2 Facility of Epidemiology and Biostatistics, Fondazione Policlinico Universitario A. Gemelli IRCCS, 00168 Rome, Italy; diana.giannarelli@unicatt.it; 3 L’Institut Du Sein-The Paris Breast Centre, 75017 Paris, France; jenny.millochau@idsein.fr (J.C.M.); marina.parapini@gmail.com (M.L.P.); 4 UOC di Radioterapia Oncologica, Dipartimento di Diagnostica per Immagini, Radioterapia Oncologica ed Ematologia, Fondazione Policlinico Universitario A. Gemelli IRCCS, 00168 Rome, Italy; fabio.marazzi@policlinicogemelli.it; 5 UOC di Oncologia Medica, Dipartimento di Scienze Mediche e Chirurgiche, Fondazione Policlinico Universitario A. Gemelli IRCCS, 00168 Rome, Italy; armando.orlandi@policlinicogemelli.it (A.O.); antonella.palazzo@policlinicogemelli.it (A.P.); 6 UOSD di Medicina Personalizzata in Senologia, Dipartimento di Scienze della Salute della Donna e del Bambino e di Sanità Pubblica, Direzione Scientifica, Fondazione Policlinico Universitario A. Gemelli IRCCS, 00168 Rome, Italy; alessandra.fabi@policlinicogemelli.it; 7 Istituto di Semeiotica Chirurgica, Università Cattolica del Sacro Cuore, 00168 Roma, Italy

**Keywords:** neoadjuvant chemotherapy, breast cancer, locally advanced breast cancer, minimally invasive treatment, oncoplastic surgery, breast cancer minimally invasive treatment

## Abstract

**Background:** Oncoplastic surgery (OPS) reliability in the post-neoadjuvant chemotherapy (NACT) setting is still debated due to weak scientific evidences in such scenarios. **Methods:** Our analysis aims to report results obtained in a retrospective series of 111 patients consecutively treated with level II OPS after NACT at the Multidisciplinary Breast Center of the Fondazione Policlinico Universitario Agostino Gemelli IRCCS between 1998 and 2018. The surgical endpoints were the mean specimen volume, rates of positive margins (PMR), re-excision (RR), conversion to mastectomy (CMR), and complications (CR). The oncological endpoints were overall survival (OS), disease-free survival (DFS), and local recurrence (LR). To evaluate the impact of NACT on surgical and oncological outcomes at 302 months, we conducted a propensity score matching, pairing patients in post-NACT and upfront surgery groups. **Results:** The mean sample volume was 390,796 mm^3^. We registered a 3.6% of PMR, 1.8% RR, 0.9% CMR, 5% CR. The 10-year OS and 10-year DFS with a median follow-up of 88 months (6–302) were 79% and 76%, respectively, with an LR recurrence rate of 5%. The post-NACT group received significantly larger excised volumes and lower PMR. NACT did not affect surgical and oncological outcomes. **Conclusions:** Level II OPS can be considered a reliable alternative to mastectomy even in the post-NACT setting.

## 1. Introduction

The widespread implementation of mammographic screening campaigns and the increased public awareness regarding breast cancer (BC) resulted in an earlier detection and fewer involved axillary nodes at the time of initial presentation.

As a result, we observed a progressive reduction in locally advanced (LABC) cases at the primary diagnosis, intended as “large operable cases” (cT3;N0–1) and “truly inoperable cases”, with thoracic wall/skin involvement (cT4a/b), supraclavicular/internal mammary nodal involvement (cN2-3), or inflammatory breast cancer cases (IBC) [1].

Nevertheless, data reported in the CONCORD high-resolution study indicate that approximately 8.5% of American and 4% of European patients still present with LABC [2], estimates that can reach up to 90% newly diagnosed BC in low- to middle-income countries [3].

In such a delicate setting, current guidelines advise for neoadjuvant chemotherapy (NACT) as primary treatment [1], especially for subtypes where the need for systemic therapy is mandatory in the adjuvant setting, such as human epidermal growth factor receptor 2 (HER2)-positive and triple-negative breast cancers (TNBC) [1,4,5].

NACT rationale, as opposed to adjuvant treatment, is to provide a systemic protection while downsizing the breast involvement, thus making operable patients who were considered inoperable at the time of diagnosis or candidates for conservative treatment (BCS) patients who initially would have received mastectomy as the only possible treatment, with conversion rates ranging from 30 to 70% [6,7,8,9,10,11].

In this particular scenario, the indications for conservative surgery could be further widened by the use of ‘oncoplastic surgery’ techniques (OPS) that combine oncological and plastic surgery procedures, which have been shown to provide wider excisions, a more efficient local control, and greater respect for aesthetic results, even for larger excisions [12].

This might suggest a significant decrease in mastectomy rates. However, it does not translate into a progressive increase in BCS rates, as reported by some European [13,14] and North American series [15,16,17].

This shortcoming can be attributed to several factors, mainly related to the weak scientific evidence about the reliability of OPS in the post-NACT setting [18].

Hence, the endpoint of our study is to report long-term results in terms of overall/disease-free survival and local control in a retrospective series of 111 patients consecutively treated with level II OPS after NACT at the Multidisciplinary Breast Center of the Fondazione Policlinico Universitario Agostino Gemelli IRCCS in Rome between 1998 and 2018. As such, this study is one of the few to evaluate the aforementioned outcomes in the post-NACT setting and the first to present a large sample analysis with a medium follow-up of more than 10 years.

## 2. Materials and Methods

From the prospectively maintained database of the Multidisciplinary Breast Center of the Fondazione Policlinico Universitario Agostino Gemelli IRCCS in Rome, we identified patients with LABC (cT1/2-cN1/2 or T3 patients, cN0-cN1/2) who received NACT and subsequent level II OPS, as previously described [19].

### 2.1. Clinical Workout

The indication for NACT (chemotherapy or endocrine therapy) and surgical management were discussed during a multidisciplinary meeting (MDM) by breast surgeons, medical oncologists, radiation oncologists, radiologists, pathologists, and geneticists.

According to national and international guidelines [1,5], patients underwent NACT in the following cases:

- Patients with LABC;
- Patients with operable breast cancer and an unfavorable breast volume/tumor size ratio in order to reduce the tumor diameter and achieve a conservative treatment instead of mastectomy;
- Patients with operable breast cancer and clinically involved lymph nodes (cN+) to ensure a sentinel lymph node biopsy (SLNB) instead of a direct axillary lymph node dissection (ALND);
- Young patients with unfavorable risk factors (Triple negative tumor, HER2+, high Ki-67 rates) to provide prompt systemic treatment.

Among these conditions, we decided to include in the present study only patients who underwent NACT for unfavorable breast volume/tumor size ratio in order to reduce the tumor diameter and achieve a conservative treatment instead of mastectomy.

#### 2.1.1. Pre-Neoadjuvant Clinical Staging

Locoregional staging was assessed with clinical examination, breast and axillary ultrasound (US), mammography (MX), breast magnetic resonance (MRI), and core biopsy of both breast lesion and suspected axillary lymph nodes, when identified.

The systemic staging was assessed via total body computed tomography scan or positron emission tomography and bone scintigraphy.

#### 2.1.2. Neoadjuvant Regimens

NACT regimen depended on stage and tumor characteristics.

HER2-negative patients:

- Sequential taxane and anthracycline-containing regimen: Anthracyclines plus Cyclophosphamide on day 1 every 21 days for 4 cycles (4 AC), followed by docetaxel on day 1 every 21 days for 4 cycles or paclitaxel on day 1 every week for 12 cycles;
- TAC: Docetaxel plus Doxorubicin plus Cyclophosphamide on day 1 every 21 days for 6 cycles.

HER2-positive patients:

- TCH: Docetaxel plus Carboplatin plus Herceptin on day 1 every 21 days for 6 cycles;
- Sequential regimen taxane and anthracycline-containing regimen + H: Anthracyclines plus Cyclophosphamide on day 1 every 21 days for 4 cycles (4 AC), followed by docetaxel on day 1 every 21 days for 4 cycles or paclitaxel on day 1 every week for 12 cycles plus Herceptin on day 1 every 21 days for 18 cycles;
- Hormone therapy: Aromatase inhibitor delivered in elder and fragile postmenopausal patients with locally advanced breast cancer expressing hormone receptors (ER, PgR) and low Ki-67 (Luminal A and Luminal B); neoadjuvant protocol administered for at least six months.

#### 2.1.3. Clinical Assessments during and after NACT

Before each cycle of chemotherapy, patients received a clinical examination and “in office” breast/axillary US.

Patients with no evidence of clinical response or with disease progression were multidisciplinary and discussed regarding a change in NACT scheme or immediate surgery.

One month after NACT finalization locoregional staging was repeated (clinical examination, breast, and axillary US, MX, MRI).

#### 2.1.4. Breast Surgical Treatment

Surgical management was discussed during a dedicated MDM, taking into account the clinical restaging and patient’s preferences.

Patients with partial response to NACT that reached a favorable ratio between breast volume and residual lesion were addressed to level II OPS when the required excision involved >20% of the entire breast volume.

In case of unfavorable ratio between breast volume and residual tumor size, multicentric localizations, inflammatory cancer, and contraindications to adjuvant radiotherapy patients were judged eligible for mastectomy and immediate breast reconstruction (implant or autologous).

Patients with complete response (intended as the absence of pathological contrast enhancement foci at MRI or visible residual lesions at MX and US) received level II OPS to excide the residual fibrosis/architectural distortion of the tumor bed if identified at the post-NACT radiological assessments.

For resections of 20–50% of the breast volume, level II OPS techniques were adopted, including “inverted T” or “J” mammoplasty, “Round Block”, central quadrantectomy with the “Grisotti” approach, and quadrantectomies with the “Batwing” techniques, according to previously described studies [12,20,21].

In all patients, a contralateral breast symmetrization was also performed in order to optimize the esthetic result. The technique used was always the same as the side of the tumor affected breast except for the Grisotti, in which a J-mastoplasty was preferred for symmetry of scars.

#### 2.1.5. Axillary Assessment

SLNB was performed only in patients that were cN0 at diagnosis or who became ycN0 after NACT, using blue dye technique (Patent Blue V or Methylene blue, 2–5 cc) as previously reported [22]. Patients with micro/macrometastatic disease received ALND.

#### 2.1.6. Adjuvant Treatment

They were decided on the basis of patient’s age, pre-neoadjuvant clinical staging, surgical intervention, pathological staging, and tumoral biology.

#### 2.1.7. Adjuvant Chemotherapy

Patients who did not reach a pathological complete response to NACT were treated according to different adjuvant regimens:

- Antracyclines and/or taxanes were given to patients who did not receive them in the neoadjuvant regimen;
- Triple-negative patients were given Capecitabine;
- HER2-positive cancers were treated with TDM-1;
- Cancers expressing hormone receptors (ER, PgR) were treated with selective estrogen receptor modulators (Tamoxifen) + or − LHRH analogues (Enantone, Decapeptyl) if in premenopausal age. Postmenopausal patients were given aromatase inhibitors (Anastrozole, Letrozole, Exemestane).

#### 2.1.8. Adjuvant Radiotherapy

It was modulated on the type of surgical intervention and pathological staging. Radiation was delivered using 3D conformal schemes and intensity modulated radiotherapy on linear accelerator using 6-10-15 MV photons.

Axillary radiation was considered for patients with pathologically positive lymph nodes and subsequent ALND with less than 10 nodes removed, ypN3 tumor staging, extracapsular invasion, or isolated tumor cells (ITC) in SLNs.

#### 2.1.9. Exclusion Criteria

- Patients with cN2 stage or more at diagnosis;
- Persistence of clinical axillary nodal metastasis at post-NACT restaging:
- Patients that presented with LABC intended as cT4 tumor;
- Metastatic patients (excluding axillary lymph nodes involvement);
- Patients treated for breast recurrent malignancy/second breast tumors;
- Patients with persistence of multifocal/multicentric disease after NACT (not intended as fragmentation of the initial lesion).

### 2.2. Endpoints and Purpose of Our Study

The purpose of this retrospective analysis was to present our results obtained with level II OPS spanning 20 years of breast cancer treatment in the post-NACT setting.

#### 2.2.1. Surgical Endpoints

- Estimates of OPS effectiveness indicators were measured during surgical procedure, intended as mean specimen volume;
- Positive Margin Rate (PMR) was defined, for invasive carcinomas, as the presence of tumor on ink, in accordance with the ASCO guidelines [23]. For cases treated until 2015 for ductal carcinomas in situ (DCIS), an involved margin was defined as tumor on ink. From 2015, an involved margin is defined as a distance lower than 2 mm between the tumor foci and the inked margin, as assessed by the European Society for Medical Oncology (ESMO) [4], and National Comprehensive Cancer Network (NCCN) guidelines [1];
- Re-excision Rate (RR) was percentage of patients undergoing surgical enlargement of margins following proven margin involvement at pathology report;
- Conversion to Mastectomy Rate (CMR) was percentage of patients who required mastectomy as the only treatment possible to obtain a margin widening following margin involvement at pathology report;
- Complications Rate (CR) was percentage of complications related to surgical treatment that occurred during the postoperative period (within 30 days from surgical procedure), including seroma, liponecrosis, hematomas, surgical site infections (including clinical signs and microbiological evidence), and wound dehiscence that required surgical reintervention.

#### 2.2.2. Oncological Endpoints

- Overall Survival (OS): calculated as the difference between the time of surgery and the date of death or censored at the date of last follow-up;
- Disease-Free Survival (DFS): calculated as the difference between the time between the time of surgery and the date of relapse (locoregional or distant) or censored at the time of last evaluation;
- Local Recurrence (LR): Number of patients who developed an ipsilateral locoregional recurrence of disease (chest wall, residual gland, skin, and axillary lymph nodes) following surgical treatment out of the total number of patients included in this study.

### 2.3. Statistical Analysis

Results are expressed as mean, median, and range. Fisher exact test was used for categorical variables. A value of *p* < 0.05 was considered statistically significant. Patients receiving level II OPS techniques were paired in the two groups based on the propensity score matching technique (nearest neighbor) using cT, cN, age, molecular subtype as control variables; a caliper of 0.5; and a 1:1 ratio. Kaplan–Meier curves were used to estimate OS, DFS, and LRR curves and compared with the log-rank test. Follow-up was calculated from surgery to last observation or death. Statistical analysis was performed with SPSS version 26.0 for Windows.

## 3. Results

Between January 1998 and January 2018, 381 patients with invasive breast cancer underwent level II OPS. The median follow-up was 88 months (6–302). According to the above-mentioned inclusion criteria, we selected 111 patients undergoing level II OPS after NACT (baseline demographic and oncologic characteristics are summarized in Table 1).

All 111 patients had unilateral, predominantly uni/multifocal lesions (94 cases-85%). In 17 cases (15%), we treated multicentric tumors with foci located on adjacent quadrants.

The mean age of the patients was 47.68 years (26–70).

### 3.1. Pathological Features

We included 84 invasive ductal carcinomas (76%), 16 lobular (14%), and 11 (10%) classified in other categories (10 no special type-NST invasive carcinomas and 1 tubular carcinoma).

Only 2 cases (2%) were well-differentiated tumors (G1), while 79 (71%) were moderately differentiated (G2) and 30 (27%) were poorly differentiated (G3).

Regarding immunohistochemical classification, we recorded 25 (23%) luminal type A tumors and 37 (33%) luminal type B tumors; 31 cases (28%) were HER2-positive tumors, divided into 15 (13.5%) HER2 positive with luminal features and 16 (14.5%) HER2 positive without luminal features. Furthermore, 18 tumors (16%) were triple negative.

### 3.2. NACT Regimes

The sequential taxane and anthracycline-containing regimen was the most frequently used (48 cases—43%), followed by regimens containing Trastuzumab (25 cases—22%), TAC regimens (13 cases—12%), and others (25 cases—23%).

### 3.3. Clinical Restaging after NACT

Regarding clinical T response, we achieved a complete response in 21 cases (19%), a partial response in 78 cases (70%), and no response in 12 cases (11%). No cases of T-stage progression (with involvement of breast skin or fascia of the pectoralis major muscle) were observed.

### 3.4. Breast Surgery

Among level II OPS techniques, we performed 42 (38%) inverted T-reduction mastoplasties (ITM), of which 33 were inferior pedicle and 9 were superior pedicle, and 52 (47%) J-reduction mastoplasties (JM), 11 (10%) “round block” (RBM) and 6 (5%) “Grisotti” (GF).

The correspondence between the tumor location and selected level II OPS technique is described in Table 1.

The average sample volume was 390,796 mm^3^ (27,000–3,700,000).

### 3.5. Pathological Response to NACT

- Response on T: We observed a complete pathological response (ypT0) in 12 patients (11%), and in 5 cases (4%), we detected residual intraductal carcinoma (ypTis). Sixty-two patients (56%) had residual ypT1 tumor, and twenty-nine (26%) were ypT2, while only three patients (3%) were ypT3;
- Response on N: Among the 111 patients, 44 (40%) that were N+ at the time of diagnosis were proven ypN0 after NACT (12/111);
- Pathological complete response: Among patients proven node positive at the time of diagnosis, we observed a complete patholocal response even on T and on N in 13 (11.7%) and an ypTis, ypN0 condition in 6 patients (5.4%).

### 3.6. Surgical Outcomes

Our positive margin rate was 3.6% (4 patients). Two cases were treated by means of a surgical margin widening; one case was converted to mastectomy, and the other one received a radiotherapy boost on the tumor bed due to the patient’s refusal to surgically widen the DCIS-infiltrated margin.

Besides the patient who was converted to mastectomy, the other three patients had focal infiltration of the margins (≤2 mm). In these situations, we propose margin widening or a high-dose radiotherapy boost (16 Gy with fractionation of 2 Gy/day or 13.35 Gy with fractionation of 2.67 Gy/day).

The radiotherapy treatment was administered with a 3D technique in 70 patients and VMAT in the remaining patients.

In our institution, the treatment of the mammary gland is performed at a dose of 40.05 Gy with fractionation of 2.67 Gy/day on the entire remaining mammary gland.

The boost is reserved for young patients (<50 years old) or those with tumor grade G3 or with massive PVI.

The boost was administered sequentially to the whole breast radiotherapy treatment with a total dose of 10 Gy and fractionation of 2.5 Gy/day. In the case of a high boost, the dose was 16 Gy with a fractionation of 2 Gy/day or 13.35 Gy with a fractionation of 2.67 Gy/day.

CTV delineated as the whole breast, from the chest wall plane to the skin minus 3 mm (crop), except when skin is in target. PTV delineated as a 0.7 cm circumferential expansion over the CTV, intersected with the skin profile. Boost CTV delineated according to the site where simulation CT shows areas of alteration due to a surgical scar or areas where surgical clips have been positioned by the surgeon.

We registered a complication rate of 5% (six patients): two cases of wound dehiscence, three seromas, and one case of surgical site infection.

In the two cases of wound dehiscence, there were no clinical signs of infection (pain, erythema, purulent outflow, fever), both registered in the vertical incision of ITM. We managed the dehiscence by means of a direct closure of the wound with detached stitches, obtaining a complete consolidation of the vertical scars in seven and ten days.

Three cases of seroma were suspected in patients presenting swelling and tension on the surgical site and were confirmed via ultrasound. We performed a US-guided aspiration of the serum with a 22 Gauge needle for all three patients. In two cases, the procedure had to be repeated weekly, obtaining a complete resolution of this condition in 21 days.

The only case of infection was diagnosed 7 days following a JM procedure. The patient presented with a monolateral, heritematous sweeling and reported fever episodes (maximum temperature 38.8 °C) that she controlled by assuming non-steroidal anti-inflammatory drugs. As a standardized internal protocol, we managed such a condition with a US-guided evacuation with a 16 Gauge needle, confirming the presence of a purulent collection that was sampled and sent for microbiological testing. The patient started an immediate wide spectrum oral antibiotic and anti-inflammatory treatment and repeated outpatient medications and US-guided evacuations every 5 days. We assisted to a complete resolution of such complications in 2 weeks.

All the complications were managed on an outpatient basis without determining any delay in adjuvant treatments. None of these six patients reported a negative impact on their quality of life during follow-up.

### 3.7. Adjuvant Treatments

All patients underwent complementary radiation therapy except for the single case of conversion to mastectomy.

No perioperative mortality was observed, and postoperative complications did not delay adjuvant treatments.

### 3.8. Oncologic Outcomes

**Figure 1:**
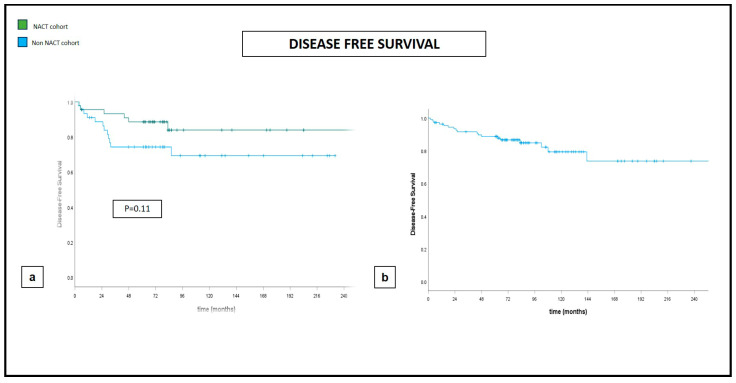
Disease-free survival at 240 months. (**a**) Comparative curves between NACT and non-NACT cohort. (**b**) Global cohort.

**Figure 2:**
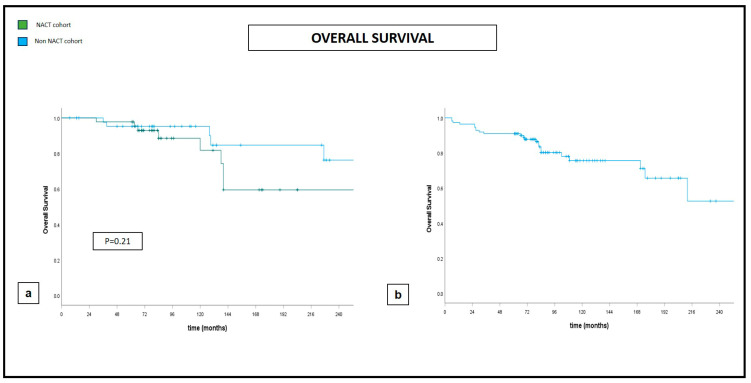
Overall survival at 240 months. (**a**) Comparative curves between NACT and non-NACT cohort. (**b**) Global cohort.

**Figure 3:**
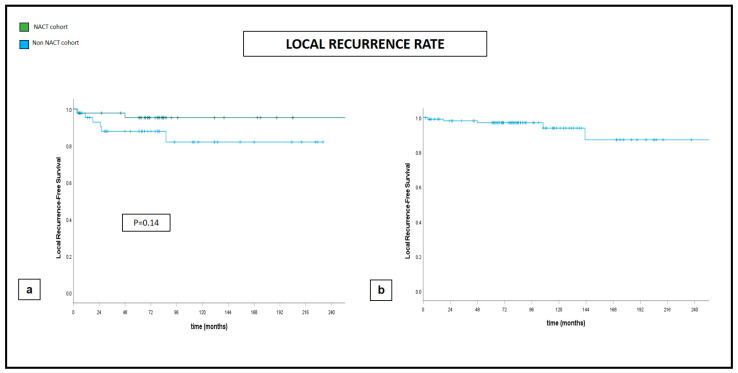
Local recurrence rate at 240 months. (**a**) Comparative curves between NACT and non-NACT cohort. (**b**) Global cohort.

Five-year and ten-year DFS and five-year and ten-year OS were 88% and 76% and 91% and 79%, respectively, with an LR rate of 3% and 5% at five and ten years, respectively (Figure 1, Figure 2 and Figure 3).

### 3.9. Matched Cohort Analysis

Even lacking any other evidence in the literature with such a long follow-up period, we performed a matched cohort analysis on a sample of 88 patients undergoing upfront post-NACT level II OPS, selected from our database on the basis of age and baseline oncological characteristics (cT, cN, molecular subtype), to evaluate the impact of NACT on surgical and oncological outcomes at 20 years.

The baseline demographic and oncologic characteristics of both groups are summarized in Table 1.

What emerges from the comparison between the two groups under analysis is a statistically significant difference (*p*-value 0.042) between the mean volumes of excised specimens. In the post-NACT group, the value is 353,439 mm^3^ (27,000–2,646,000), while in patients who underwent upfront surgery, it is 203,334 mm^3^ (217–1,275,000).

We did not observe any positive margin in the post-NACT group, whereas among patients undergoing upfront surgery, we had a PMR of 16% (7 patients).

## 4. Discussion

The foremost rationale for NACT in locally advanced breast cancer is to provide an effective systemic protection while downstaging the locoregional involvement. When the latter condition is met, patients may access conservative treatments with better cosmetic outcomes and reduced psychological impairment [24,25,26].

However, this potential remains frequently unfulfilled, as responses to NACT still do not translate into a significant reduction in mastectomies, which are offered as a primary treatment to this particular subgroup of patients [13,14,15,16,17].

Consequently, OPS remains underapplied in this setting, where its potential could instead increase BCS rates, ensuring high standards of oncologic safety (as reported by Losken and De La Cruz meta-analyses) [25,26] and respect for cosmetic and psychological outcomes.

In our experience, with the use of NACT, we observed rates of clinical downstaging and a pathologic complete response of 70% and 11%, consistent with previously reported data [8,9,10,11,27,28].

With these response rates and the use of level II OPS techniques, we reached the excision of considerable glandular volumes (390 cm^3^), wider than those reported by Mazouni, who reported excisions of 180 cm^3^ [29].

This phenomenon is mostly due to our post-NACT surgical planning, which was based on image processing protocols for an accurate preoperative localization [30,31], leading to the excision of residual neoplastic foci along with the fibrosis that arises as a clinical response to treatment, when detected. In none of the reported cases, the overall extent of excided volume corresponded dimensionally to the original tumoral extension, but it was variably reduced according to the degree of NACT response.

This multidisciplinary workout led us on one hand to perform significantly larger excisions than those in the non-NACT group (353 cm^3^ versus 203 cm^3^) but on the other hand to achieve lower rates of involved margins and a lower need for re-excision (0% versus 16% and 0% versus 9%, respectively), without any recorded cosmetic penalization or psychosocial disadvantage.

The overall complication rate was 5%, in line with data reported by Mazouni (9%) [29] but significantly lower than the events reported by Adamson, Gulcelik, and Emiroglu (23%, 28%, and 16.7%, respectively) [31,32,33].

However, the latter studies took into account minimal imperfections in the healing process, such as the delayed consolidation of the surgical wound or liponecrotic areas not requiring surgical treatment [31,32,33].

In our clinical practice, these factors are not recorded as complications but simply as situations requiring outpatient solutions (such as advanced dressings or percutaneous ultrasound-guided evacuation).

As far as we know, our experience is the first to report level II OPS oncological outcomes with a median follow-up of 88 months. Therefore, we matched our OS, DFS, and LRR rates at 60 months with previously reported data: our OS and DFS rates are 91% and 88.8%, respectively, in line with those reported by Mazouni (OS 96.2% and DFS 92.7%) [29] or Gucelik (OS 92% and DFS 90.1% at 5-years) [31].

Conversely, Da Costa Vieira and Clough with the Paris Breast Centre reported significantly lower OS and DFS rates (81.7–6.5% and 85.3–67.4%, respectively). The first was due to the inclusion of 26.9% cT4 cases [34], and the latter was due to the inclusion of patients with larger lesions at diagnosis (mean initial radiological tumor size was 45.7 mm) and high axillary involvement (9.2% of patients with more than four metastatic lymph nodes at diagnosis) [35].

Regarding 5-year LRR, we observed a 2.9% rate, aligning with recent meta-analyses that analyzed these results under more favorable conditions (patients who did not receive NACT), which reported 4.7% and 3.2%, respectively [25,26].

The same comparison is not possible with previously reported data in the NACT setting because of extremely heterogeneous results, ranging from 0% at 60 months reported by Clough (with a PMR of 26% and need for reintervention in 18%) [35] to 14.6% at 61 months by Emiroglu (with a PMR of 7.3% and reintervention rate of 7.1%) [32].

Our propensity score matching documented significantly larger excised volumes and significantly lower positive margin rates in the post-NACT group of patients. There was no impact given by NACT on either the development of postoperative complications or distant oncologic outcomes, intended as OS, DFS, and LR, though the sample size could not allow for definitive conclusions (Figure 1, Figure 2 and Figure 3).

## 5. Conclusions

Despite the limitations of its retrospective nature, our results confirmed these concluding remarks:

- Level II OPS can be considered a reliable alternative to breast conserving surgery or demolitive procedures, such as conservative mastectomies, in cases with a glandular demolition range of 20–30% of the overall glandular volume, even in the post-NACT setting. Our surgical and oncological outcomes strengthen the reported data, mostly based on lower sample sizes and a shorter follow-up period;
- Breast surgeons must be fully trained in level II OPS, and these techniques have to be more frequently offered to patients who benefit from a NACT response in the conviction that this treatment is not only reliable, but it also can grant a more natural result;
- Our study analyzed 20 years of surgical activity in a high-volume breast unit, where the treatments offered have always been the result of multidisciplinary diagnostic and therapeutic protocols, especially in the post-NACT setting. The implementation of level II OPS techniques in such a delicate scenario can only take place in highly trained centers.

## Figures and Tables

**Table 1:** Baseline demographic and oncologic characteristics of patients.

	Entire NACT Cohort (111 Patients)	Post-NACT Matched Cohort (44 Patients)	Non-NACT Matched Cohort (44 Patients)	*p*-Value
**Mean age**	47.68 (26–70)	46.53 (30–65)	47.46 (24–72)	0.50
**Histology**				0.45
Ductal	84 (76%)	34 (77%)	33 (75%)	
Lobular	16 (14%)	8 (18%)	6 (14%)	
Other Categories	11 (10%)	2 (5%)	5 (11%)	
**Grading**				0.30
1	2 (2%)	2 (5%)	7 (16%)	
2	79 (71%)	26 (59%)	26 (59%)	
3	30 (27%)	16 (36%)	11 (25%)	
**Luminal Type**				0.31
Luminal A	25 (23%)	10 (23%)	10 (23%)	
Luminal B	37 (33%)	12 (27%)	7 (16%)	
HER2+ luminal	15 (13.5%)	7 (16%)	15 (34%)	
HER2+ non luminal	16 (14.5%)	5 (11%)	5 (11%)	
Triple-negative	18 (16%)	10 (23%)	7 (16%)	
**cT stage**				0.35
1	23 (21%)	10 (23%)	5 (11%)	
2	72 (65%)	28 (64%)	33 (75%)	
3	16 (14%)	6 (13%)	6 (14%)	
**cN stage**				0.49
0	67 (60%)	29 (66%)	32 (73%)	
1	44 (40%)	15 (34%)	12 (27%)	
**NACT regimens**				
Sequential	48 (43%)	23 (52%)	-	
Herceptin containing regimen	25 (22%)	1 (2%)	-	
TAC	13 (12%)	3 (7%)	-	
Others	25 (23%)	17 (39%)	-	
**Clinical Response on T**				
Complete	21 (19%)	10 (23%)	-	
Partial	78 (70%)	30 (68%)	-	
No response	12 (11%)	4 (9%)	-	
**Upper quadrants**	68 (61%)	22 (50%)	26 (59%)	
Inverted T mammoplasty with inferior pedicle	30 (44%)	12 (55%)	15 (58%)	
J- reduction mammoplasty	31 (46%)	9 (41%)	8 (31%)	
Round block technique	7 (10%)	1 (4%)	3 (11%)	
**Central quadrants**	11 (10%)	5 (11%)	-	
J-reduction mammoplasty	4 (36%)	1 (20%)	-	
Round block	3 (28%)	2 (40%)	-	
Grisotti	4 (36%)	2 (40%)	-	
**Lower quadrants**	15 (14%)	7 (16%)	11 (25%)	
Inverted T mammoplasty with superior pedicle	8 (53%)	4 (57%)	6 (55%)	
J-**reduction** mammoplasty	7 (47%)	3 (43%)	4 (36%)	
Round block technique	-	-	1 (9%)	
**Multicentric tumors**	17 (15%)	10 (23%)	7 (16%)	
Inverted T mammoplasty with inferior pedicle	3 (18%)	1 (10%)	4 (57%)	
Inverted T mammoplasty with upper pedicle	1 (6%)	-	3 (43%)	
J-reduction mammoplasty	10 (58%)	8 (80%)	-	
Round block technique	1 (6%)	1 (10%)	-	
Grisotti technique	2 (12%)	-	-	
**Mean specimen volume (mm^3^)**	390,796 (27,000–3,700,000)	353,439 (27,000–2,646,000)	209,921 (217–1,275,000)	0.042
**ypT**				0.001
0	12 (11%)	5 (11%)	-	
Is	5 (4%)	1 (2%)	2 (5%)	
1	62 (56%)	25 (57%)	13 (29%)	
2	29 (26%)	12 (28%)	27 (61%)	
3	3 (3%)	1 (2%)	2 (5%)	
**ypN**				0.42
0	36 (32%)	22 (50%)	21 (47%)	
1	48 (43%)	15 (34%)	19 (43%)	
2	24 (22%)	6 (14%)	2 (5%)	
3	3 (3%)	1 (2%)	2 (5%)	
**Complications Rate**	6 (5%)	2 (5%)	3 (7%)	1.000
Wound dehiscence	2 (33%)	1 (50%)	1 (33%)	
Seroma	3 (50%)	1 (50%)	1 (33%)	
Infection	1 (17%)	-	-	
Hematoma	-	-	1 (33%)	
Skin necrosis	-	-	-	
**Positive Margin Rate**	4 (3.6%)	-	7 (16%)	0.012
Re-excision Rate	2 (50%)	-	4 (57%)	
Conversion to mastectomy rate	1 (25%)	-	-	
Boost	1 (25%)	-	3 (43)	
**Adjuvant therapy**				
Hormone therapy	72 (65%)	29 (66%)	26 (59%)	0.660
RT	110 (99%)	44 (100%)	44 (100%)	1.000
**10-year Overall survival**	79%	89%	95%	0.207
**10-year Disease-Free Survival**	76%	84%	69%	0.106
**10-year Local recurrence**	5%	5%	18%	0.140

## Data Availability

Data are contained within the article.
